# A Physical Framework to Interpret the Effects of High Permittivity Materials on Radiofrequency Coil Performance in Magnetic Resonance Imaging

**DOI:** 10.1109/TBME.2022.3165763

**Published:** 2022-10-19

**Authors:** Giuseppe Ruello, Riccardo Lattanzi

**Affiliations:** Department of Electrical Engineering and Information Technology, University of Napoli Federico II, 80125 Naples, Italy; Center for Advanced Imaging Innovation and Research (CAI2R) and the Bernard and Irene Schwartz Center for Biomedical Imaging, Department of Radiology, New York University Grossman School of Medicine, USA.

**Keywords:** High permittivity materials, electromagnetic modeling, electromagnetic propagation, magnetic resonance imaging

## Abstract

**Objective:**

We propose a framework to interpret the effects of High Permittivity Materials (HPM) on the performance of radiofrequency coils in Magnetic Resonance Imaging (MRI).

**Methods:**

Based on a recent formulation of the scattering and propagation properties of spheres, we expanded the field in a layered sphere as a superposition of ingoing and outgoing travelling waves, which allowed us to study the field components with a non-homogeneous transmission line model. We investigated the effects of a layer of HPM surrounding a head-mimicking uniform sphere at 7 tesla.

**Results:**

Through the analysis of impedance and reflection coefficients, we show that by adjusting the properties of the HPM, it is possible to selectively amplify individual modes, or combination of them, modifying the overall field distribution in the sample and increasing signal-to-noise ratio at specific locations. Our results demonstrate that the observed enhanced MRI performance is not merely due to secondary fields generated by displacement currents in the HPM.

**Conclusions:**

Our formulation explains the effect of HPM in terms of matching, enabling the optimization of the electrical properties of the HPM with a simple mode-matching formula.

**Significance:**

The proposed method could guide the design of novel radiofrequency coils with integrated HPM.

## I. Introduction

Ultra-High Field (UHF) Magnetic Resonance Imaging (MRI), which refers to the use of MR scanners with static magnetic field strength greater than 3 tesla, has been one of the most promising innovation trends in diagnostics [1]–[5]. The enormous potential of UHF MRI has been understood since the first prototypes were developed in the 1990s, but its full exploitation remains far from being achieved [1], [4], [6].

The main constraints in UHF MRI are related to the interactions of the radiofrequency (RF) fields with the human body, due to the centimetric wavelengths associated with the high Larmor frequency (e.g., 297.2 MHz at 7 tesla). In fact, these interactions result in transmit $\left(B_{1}^{+}\right)$ and receive $\left(B_{1}^{-}\right)$ field inhomogeneity, which can cause image artifacts and potentially harmful local specific RF power absorption rate (SAR) hotspots in the exposed tissues [1], [7].

While it has been shown that parallel transmission with multiplie RF coils could mitigate inhomogeneities and control SAR [8]–[11], such solution is expensive and leads to longer exams. Furthermore, not all UHF systems are equipped with multiple transmit channels and there are not many commercial RF coils available with multiple transmit elements. For these reasons, alternative approaches that can be implemented with standard MR hardware have been explored.

For example, it was shown that the use of High Permittivity Materials (HPM) in the coil-tissue system could help improving field homogeneity, signal-to-noise ratio (SNR) and SAR in UHF MRI [12]–[17]. Previous works with HPM is mostly based on empirical or experimental approaches, which do not provide a clear and comprehensive interpretation of the physical phenomena yielding enhanced MRI performance. As a result, such effect is often associated with the creation of secondary magnetic fields by displacement currents inside the HPM, which contribute to the received MR signal or the transmitted RF field [15], therefore increasing SNR and improving transmit efficiency. However, this analysis fails to explain why these secondary magnetic fields prevail over the secondary electric fields, which are equally generated by the same displacement currents but negatively affect coil performance, by contributing to the received noise or the RF power dissipation. While a simulation framework has been recently proposed to explain how HPM can improve SNR via qualitative observations of ideal current patterns [16], an electrodynamic analysis to shed light on the fundamental mechanisms of interaction between RF fields and HPM has not been performed yet.

To address this, we propose an analytical framework for the study of electromagnetic (EM) field propagation in HPM based on a recently published formulation of the scattering from spheres [18]. In such framework, the field is described as a superposition of elementary waves (modes), which are expressed in terms of spherical travelling waves in every layer of the sphere. Mode superposition has been employed to represent the EM field in spheres in both classical and recent literature [19]–[22]. Following this approach, one can model field propagation using non-uniform transmission lines. The formulation introduced by [18] has enriched the established work on non-uniform transmission lines [23]–[27] by providing a simple formula for the impedance transport that allows one to express reflection and transmission coefficients in terms of the layered sphere impedances, enabling a straightforward engineering analysis. Here, we employ this formulation to investigate a system composed of a uniform sphere surrounded by a layer of HPM. The HPM-sphere system was placed in air and illuminated by a source of spherical waves. Note that using a spherical model with tissue-mimicking electrical properties as a first order approximation of a human head is not new in MRI and already provided reliable physical insight [28], [29].

The RF field distribution in the sphere is affected by the current density on the coil conductors, the distance of the coil from the sphere, the geometry and properties of the sphere, as well as the thickness, dielectric constant, and conductivity of the HPM layer. Previous work was mostly based on numerical or experimental approaches, from which it is not possible to isolate the effect due to the HPM layer from all these other contributions. In this study, we were able to investigate the role of HPM separately, by observing that the HPM layer influences only the radial dependence of the RF field. This allowed us to separate the field propagation and study only the factors affected by the HPM. We show that, for a given thickness of the HPM layer, our proposed approach allows one to estimate the optimal value of the HPM permittivity that maximizes the amplitude of individual modes. As a result, it is possible to shape the field inside the sample by designing the HPM to selectively amplify specific modes and optimize MRI performance based on the diagnostic goal.

## II. Theory

### A. Electrodynamic Model

In this section, we recall the basic principles of a recently introduced formulation of the scattering from spheres [18]. In the following, we will consider only the Transverse Electric (TE) polarization. An equivalent analysis and comparable results can be obtained also for the Transverse Magnetic (TM) polarization, but are not presented here for the sake of brevity.

The electric and magnetic transverse fields in the *l*^th^ medium of a layered sphere can be expressed as a superposition of ingoing and outgoing modes:

(1a) $${\text{E}}_{l}(r)=\sum_{n=1}^{\infty }\sum_{m=-n}^{n}E_{lnm}^{+}{\text{M}}_{nm}^{\left(3\right)}+E_{lnm}^{-}{\text{M}}_{nm}^{\left(4\right)}$$

(1b) $${\text{H}}_{l}(r)=\frac{k}{i\omega \mu }\sum_{n=1}^{\infty }\sum_{m=-n}^{n}E_{lnm}^{+}{\text{N}}_{nm}^{\left(3\right)}+E_{lnm}^{-}{\text{N}}_{nm}^{\left(4\right)}$$

where $E_{lnm}^{+}$ and $E_{lnm}^{-}$ are the coefficients of the ingoing and outgoing modes, respectively. The modes are expressed in terms of the vector harmonics **M**_*nm*_ and **N**_*nm*_:

(2a) $${\text{M}}_{nm}=B_{n}(kr)\left[i\pi_{nm}(\vartheta )\hat{\vartheta }-\tau_{nm}(\vartheta )\hat{\varphi }\right]e^{im\varphi }$$

(2b) $${\text{N}}_{nm}=\frac{1}{kr}\frac{d\left(rB_{n}(kr)\right)}{dr}\left[i\pi_{nm}(\vartheta )\hat{\varphi }+\tau_{nm}(\vartheta )\hat{\vartheta }\right]e^{im\varphi }\;\;\;\;\;\;\;\;\;\;\;\;\;\;+\frac{B_{n}(kr)}{kr}n(n+1)P_{n}^{m}(\cos\vartheta )e^{im\varphi }\hat{r}$$

where *i* is the imaginary unit, (r,ϑ,φ) are the three coordinates of a spherical coordinate system with the origin in the center of the sphere, *B*_*n*_ is a spherical Bessel function of the *n*^*th*^ order, ${\text{P}}_{n}^{m}$ is the associated Legendre polynomial, and *π*_*nm*_ and *τ*_*nm*_ are sectorial functions [18]:

(3a) $$\pi_{nm}(\vartheta )=m\frac{{\text{P}}_{n}^{m}(\cos\vartheta )}{\sin\vartheta }$$

(3b) $$\tau_{nm}(\vartheta )=\frac{d{\text{P}}_{n}^{m}(\cos\vartheta )}{d\vartheta }.$$

Note that the modes are separated and the dielectric properties of each layer affect the corresponding propagation constant *k*, which is only accounted for in the radial term *B*_*n*_(*kr*). Therefore, the radial dependence of the field is sufficient to investigate the effects of the HPM layer on the field propagation. The term *B*_*n*_(*kr*) can be written as a combination of static spherical Bessel functions of the first and second kind, or traveling spherical Bessel functions of the third and fourth kind (also called spherical Hankel functions of the first and second kind). It was shown that the transverse fields propagating in each layer can be described by travelling and stationary fields with a formalism that recall the transmission line theory [18].

In particular, in the *l*^th^ medium, for the *n*^th^ mode, the transverse electromagnetic fields can been expressed in compact form as [18]:

(4a) $$E_{ln}=E_{ln}^{+}h_{n}^{\left(1\right)}\left(k_{l}r\right)\left[1+\Gamma_{n}\left(k_{l}r\right)\right]$$

(4b) $$H_{ln}=\frac{E_{ln}^{+}}{Z_{nl}}h_{n}^{\left(1\right)}\left(k_{l}r\right)\left[1+\Gamma_{n}\left(k_{l}r\right)\frac{Z_{nl}}{\bar{Z_{nl}}}\right].$$

In equations (4), $h_{n}^{\left(1\right)}$ is the spherical Bessel function of the first kind, $Z_{nl}$ and $\bar{Z_{nl}}$ are the impedances of the ingoing and outgoing waves defined in Table I, and Γ_*n*_(*k*_*l*_*r*) is the reflection coefficient defined as:

(5) $$\Gamma_{n}\left(k_{l}r\right)=\frac{E_{ln}^{-}h^{\left(2\right)}\left(k_{i}r\right)}{E_{ln}^{+}h^{\left(1\right)}\left(k_{i}r\right)},$$

where *h*^(2)^ is the spherical Bessel function of the second kind. Note that in equations (4), we exploited the fact that the field coefficients $E_{lnm}^{+}$ do not depend on the index *m* to simplify the notation and pose $E_{lnm}^{+}=E_{ln}^{+}\forall m$.

Equations (4) describe the field in each layer as the product of a geometrical term provided by the spherical Hankel functions, which accounts for the energy radiation of spherical waves, and the term [1+ Γ_*n*_(*k*_*l*_*r*)], which accounts for the interaction of ingoing and outgoing waves. As in classical transmission line theory, the reflection coefficient is linked to the concept of impedance (Table I):

(6) $$\Gamma_{n}\left(k_{l}r\right)=\frac{Z_{n}(r)-Z_{nl}}{Z_{nl}-Z_{n}(r)\frac{Z_{nl}}{Z_{nl}}},$$

where *Z*_*n*_(*r*) is the overall impedance at a distance *r* from the origin of the sphere.

The impedance at the *l*^th^ layer can be calculated as the ratio between electric and magnetic fields:

(7) $$Z_{n}(r)=Z_{Jnl}\frac{A_{0l}+it_{nl}}{A_{0l}+it_{nl}^{\prime }},$$

where

(8) $$A_{0l}=\frac{\left(E_{ln}^{+}+E_{ln}^{-}\right)}{\left(E_{ln}^{+}-E_{ln}^{-}\right)},$$

(9) $$t_{nl}=\frac{y_{n}\left(k_{l}r\right)}{j_{n}\left(k_{l}r\right)},$$

and

(10) $$t_{nl}^{\prime }=\frac{y_{n}^{\prime }\left(k_{l}r\right)}{j_{n}^{\prime }\left(k_{l}r\right)}$$

and we used the stationary form of the fields:

(11) $$E_{ln}=\left(E_{ln}^{+}+E_{ln}^{-}\right)2j_{n}\left(k_{l}r\right)+i\left(E_{ln}^{+}-E_{ln}^{-}\right)2y_{n}\left(k_{l}r\right)$$

(12) $$H_{ln}=\frac{k_{l}}{i\omega \mu }\left(E_{ln}^{+}+E_{ln}^{-}\right)2j_{n}^{\prime }\left(k_{l}r\right)+i\left(E_{ln}^{+}-E_{ln}^{-}\right)2y_{n}^{\prime }\left(k_{l}r\right)$$

The above-described model is applicable for both the case of lossless and lossy media. In the latter case, which is of particular interest in MRI because biological tissues have non-zero conductivity, the propagation constant of the innermost medium is complex and the sample impedance is not purely imaginary, as in the lossless case.

## III. Results

**Fig. 1.**
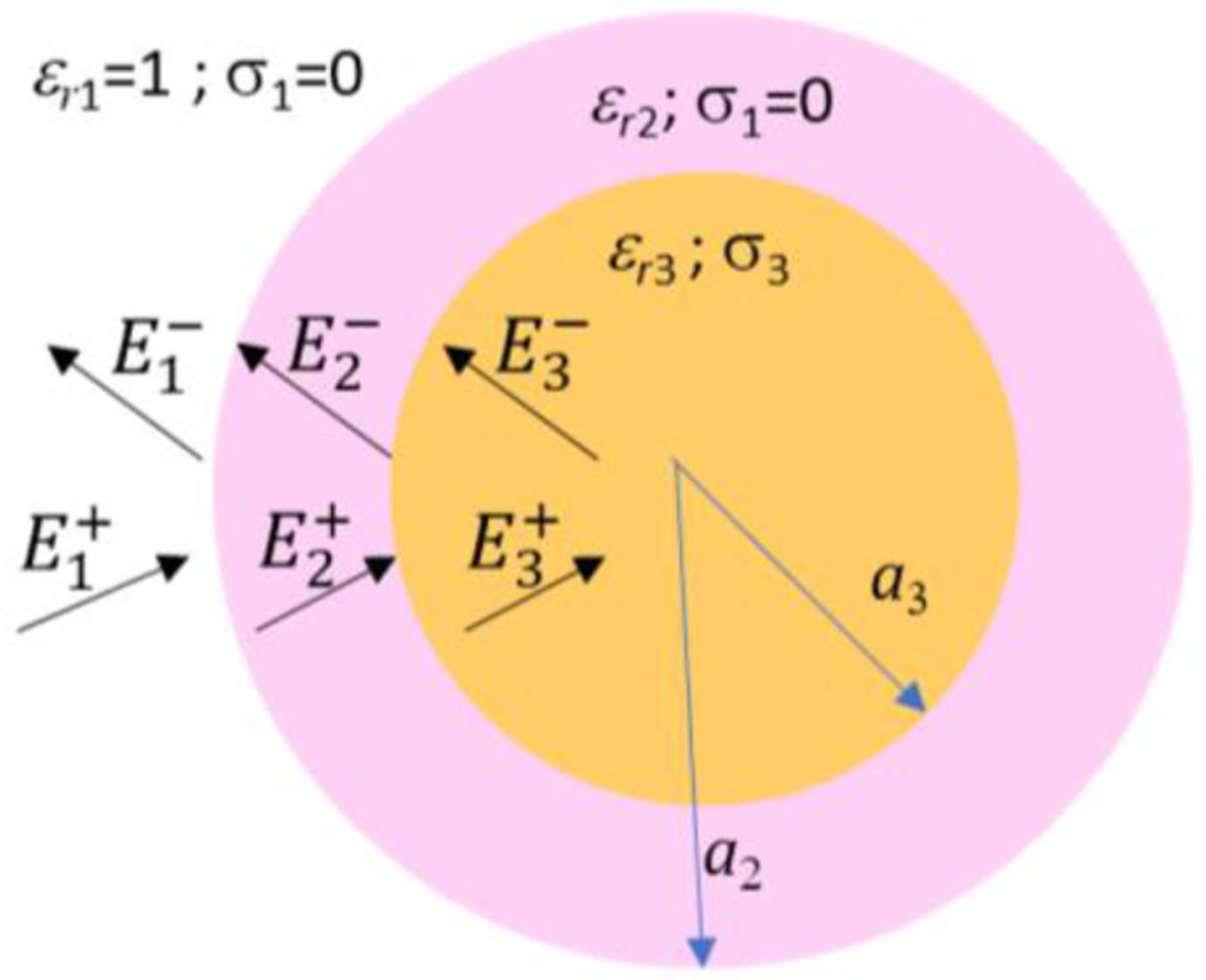
Geometry of the problem. The outer medium is air ( *ε*_*r*1_ = 1*, σ*_1_ = 0 *S/m*); the lossless HPM layer (pink) has dielectric permittivity *ε*_*r*2_; the inner medium is characterized by the dielectric parameters *ε*_*r*3_ and *σ*_3_.

In this section we investigate the effect of an external layer of HPM on the field distribution inside the spherical sample. The geometry of the problem is provided in Fig. 1, where we depicted in yellow the spherical sample (medium 3) and in pink the HPM layer (medium 2). The layered structure is surrounded by air (medium 1).

The incident field is a spherical wave travelling from the external medium to the sample. We set the wave frequency to 297.2 MHz, corresponding to the Larmor frequency of a 7 tesla MRI system, but the following can be applied to any frequency.

The overall field distribution depends on the geometrical and dielectric characteristics of the scatterer, as well as the incident field coefficients that are determined by the source of the field.

In the analysis of the results, the contributions of the source and the scatterer could mix, making it difficult to understand the specific effect of the HPM. Since the incident field can be expressed as the superposition of ingoing modes, to more easily study the contribution of the HPM alone on the field propagation, we assume that all the modes have the same unit coefficients $\left(E_{1n}^{+}=1\frac{kV}{m}\forall n\right)$. We start by presenting simple canonical cases: first, the fundamental mode and the second mode are investigated separately; then, their combination is studied to show the effect on a more realistic field distribution.

### A. Fundamental Mode (n = 1) in the Lossless Sphere

Let us begin by considering the distribution of the electric and magnetic field associated with the fundamental mode (*n* = 1) in a homogeneous lossless sphere (*σ*_3_ = 0) with dielectric permittivity *ε*_*r*3_ and radius *a*_3_ = 9 *cm*.

In absence of the HPM layer ( *ε*_*r*2_ = 1), medium 2 and medium 1 are the same, and the field coefficient $E_{31}^{+}$ of the ingoing field inside the sphere can be evaluated by imposing the continuity condition as:

(13) $$E_{31}^{+}=E_{11}^{+}\frac{h_{1}^{\left(1\right)}\left(k_{1}a_{3}\right)}{2j_{1}\left(k_{3}a_{3}\right)}\left[1+\Gamma_{1}\left(k_{1}a_{3}\right)\right]$$

where we used the condition $E_{31}^{-}=E_{31}^{+}$, derived in [18] to ensure energy conservation.

The mode coefficient in equation (13) is given by the product between a geometrical coefficient, given by the ratio of the Hankel and Bessel functions, and a transmission coefficient *τ*_13_ = 1 + Γ_1_(*k*_1_*a*_3_). From the previous equation, we see that the field peaks when the reflection coefficient Γ_1_ (*k*_1_*a*_3_) = 1, which means that the resonance condition corresponds to the ingoing and outgoing waves being in phase.

As a result, by substituting in equation (6) *r* = *a*_3_ and *k*_*l*_ = *k*_1_, we obtain that the impedance seen at the interface *a*_3_ must satisfy:

(14) $$Z_{1}\left(a_{3}\right)=\frac{2Z_{n1}\bar{Z_{n1}}}{Z_{n1}+\bar{Z_{n1}}}=i\frac{{\left|Z_{n1}\right|}^{2}}{Im\left(Z_{n1}\right)}=Z_{M}\text{.}$$

**Fig. 2.**
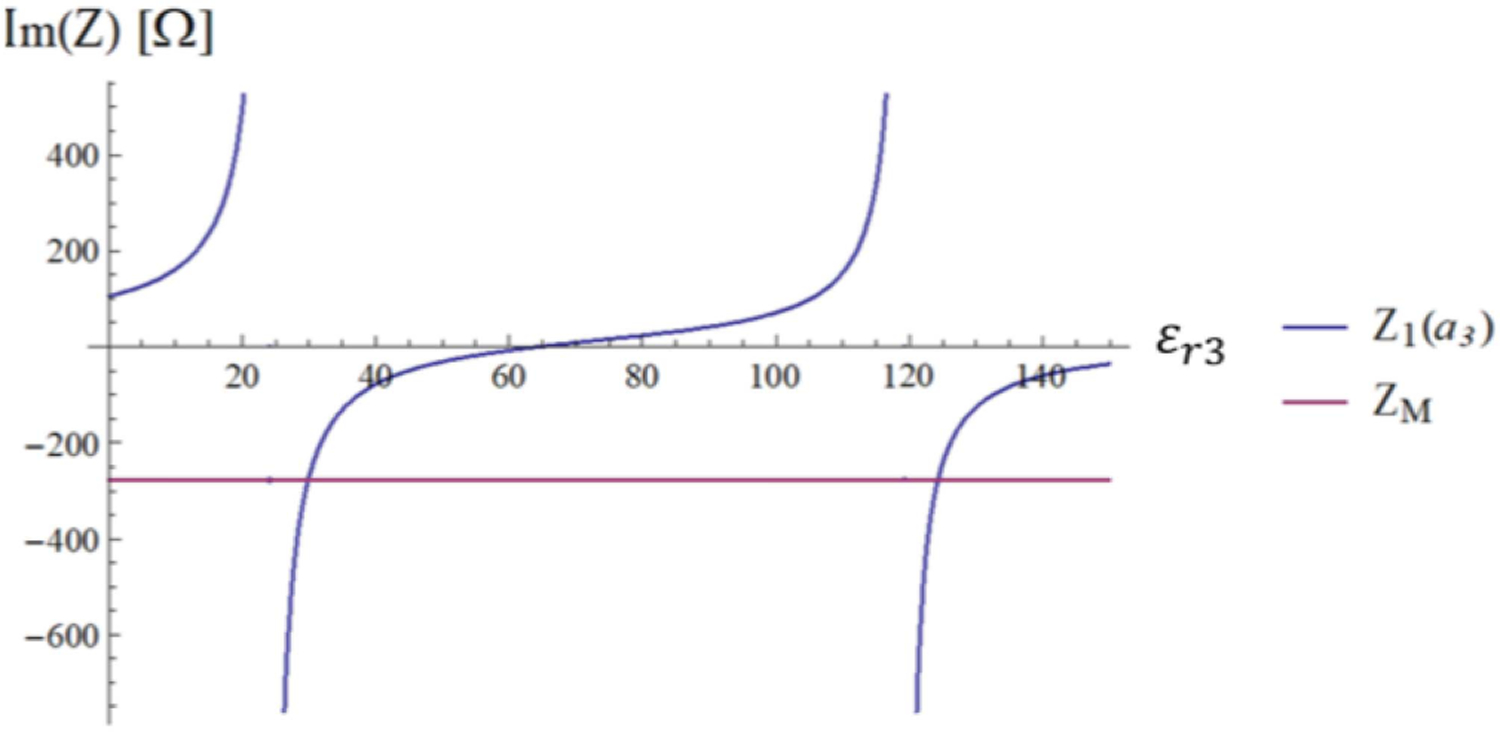
The impedance *Z*_1_(*a*_3_) seen at the sphere interface is represented in blue. The matching value defined by the second member of equation (14) is represented in magenta.

This condition is met for a countable infinity of *ε*_*r*3_ values, as graphically shown in Fig. 2. The solutions are given by the intersection of the two curves that represent the imaginary part of *Z*_1_(*a*_3_) and *Z*_*M*_ (matching impedance) as a function of *ε*_*r*3_ and identify the dielectric constants that lead to resonance.

It is worth specifying that the aforementioned phenomenon is an actual dielectric resonance, not to be confused with field amplifications due to constructive interference patters, which happen in MRI when the RF wavelength is short compared to the size of the sample [5], [30]–[32]. The above formalism enabled us to predict in a straightforward manner what values of *ε*_*r*3_ maximize the field distribution inside the homogeneous sphere.

In MRI, the *ε*_*r*3_ of the sample is obviously given, but we can nevertheless steer the electromagnetic field in the sample by surrounding the sample with an HPM layer with suitable thickness and relative permittivity. Let us assign *ε*_*r*3_ = 50 to the lossless innermost medium, which mimics the average dielectric constant of brain tissue at the frequency of interest. In the following, we fix the thickness of the HPM layer to *d* = 1.596 *cm* and investigate the effect of its relative permittivity *ε*_*r*2_ on the field distribution for the fundamental mode. We chose *d* arbitrarily to correspond to half wavelength when *ε*_*r*2_ = 1000, a value that was used for the HPM in a previous work [16].

We first impose the continuity conditions for the electric field at the two interfaces *a*_2_ and *a*_3_, which enables us to relate the field coefficients in the inner and in the outer medium as:

(15) $$E_{31}^{+}=E_{11}^{+}\frac{h_{1}^{\left(1\right)}\left(k_{1}a_{2}\right)h_{1}^{\left(1\right)}\left(k_{2}a_{3}\right)}{h_{1}^{\left(1\right)}\left(k_{2}a_{2}\right)j_{1}\left(k_{3}a_{3}\right)}\;\;\;\;\;\;\;\;\;\;\;\;\;\;\cdot \frac{\left[1+\Gamma_{1}\left(k_{1}a_{2}\right)\right]\left[1+\Gamma_{1}\left(k_{2}a_{3}\right)\right]}{\left[1+\Gamma_{1}\left(k_{2}a_{2}\right)\right]}$$

As for the previous example, also in equation (15) the coefficient of the fundamental mode is the product of a geometric factor with an electromagnetic factor, given by a combination of three transmission coefficients:

(16) $$\tau_{12}=1+\Gamma_{1}\left(k_{1}a_{2}\right),$$

(17) $$\tau_{23}=1+\Gamma_{1}\left(k_{2}a_{3}\right),$$

(18) $$\tau_{22}=1+\Gamma_{1}\left(k_{2}a_{2}\right).$$

These coefficients represent the interferences between ingoing and outgoing fields associated with the different media.

**Fig. 3.**
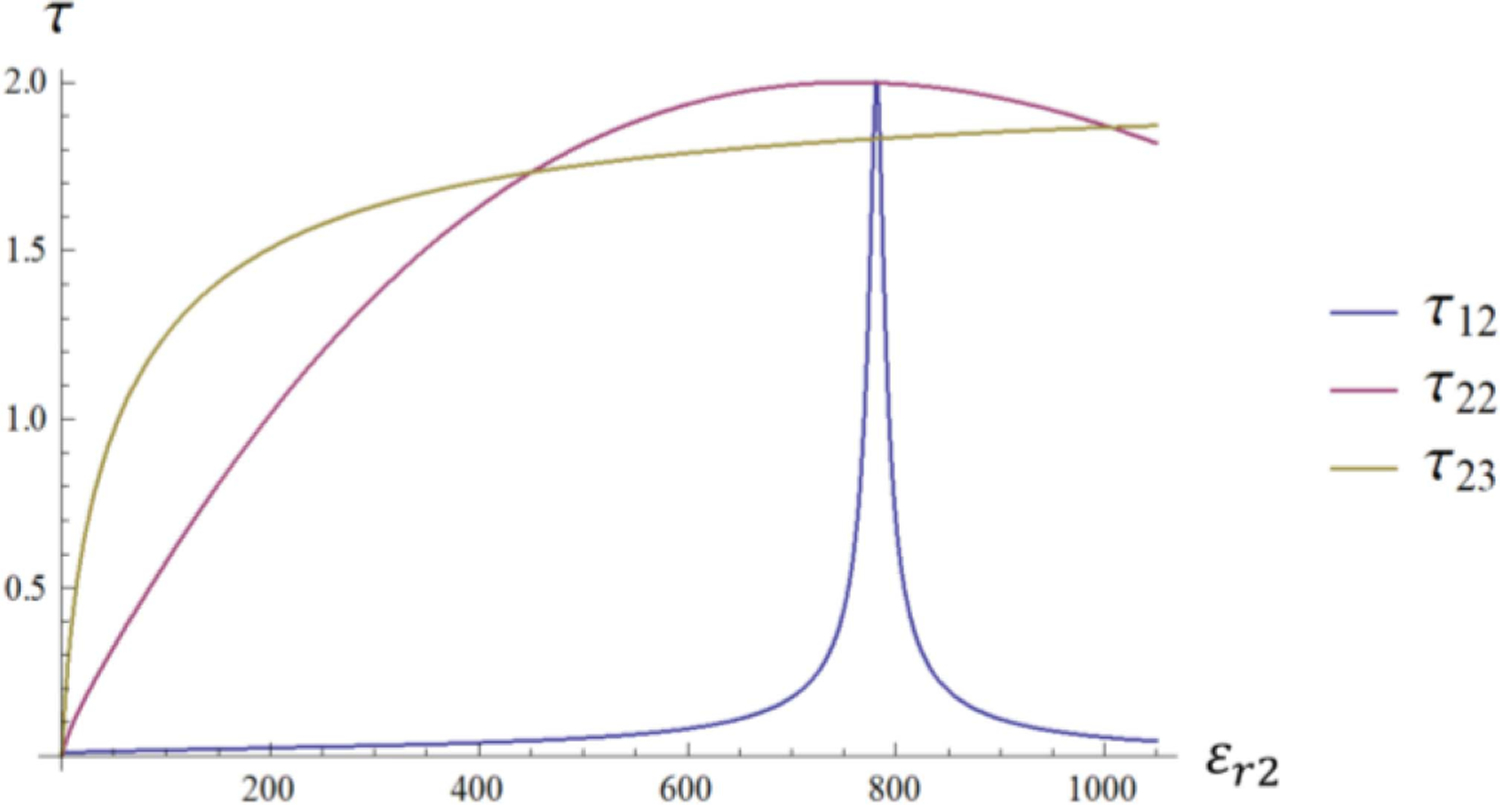
Transmission coefficients *τ*_12_ (blue line), *τ*_22_ (magenta line) and *τ*_23_ (gold line).

It is expected that the amplitude of the $E_{31}^{+}$ coefficient depends mainly on the *τ*_12_ coefficient, which describes the phase relation between ingoing and outgoing waves at the outermost layer interface. This intuition is confirmed by the plots in Fig. 3, which show that a resonance condition can be induced by setting the *ε*_*r*2_ of the HPM layer to the value for which the *τ*_12_ coefficient peaks.

The value of *ε*_*r*2_ corresponding to the peak of the field can be calculated by imposing a simple engineering condition:

(19) $$Z_{1}\left(a_{2}\right)=\frac{2Z_{n1}\bar{Z_{n1}}}{Z_{n1}+\bar{Z_{n1}}}=i\frac{{\left|Z_{n1}\right|}^{2}}{Im\left(Z_{n1}\right)}=Z_{M}\text{,}$$

where *Z*_1_(*a*_2_) is the overall impedance seen at the external interface, which can be evaluated with equation (7). Note that equation (19) admits solutions because the inner medium is lossless and, as a consequence, the impedance *Z*_1_(*a*_2_) on the left-hand-side is purely imaginary as the matching impedance *Z*_*M*_ on the right-hand-side. The case of a lossy medium is discussed later.

**Fig. 4.**
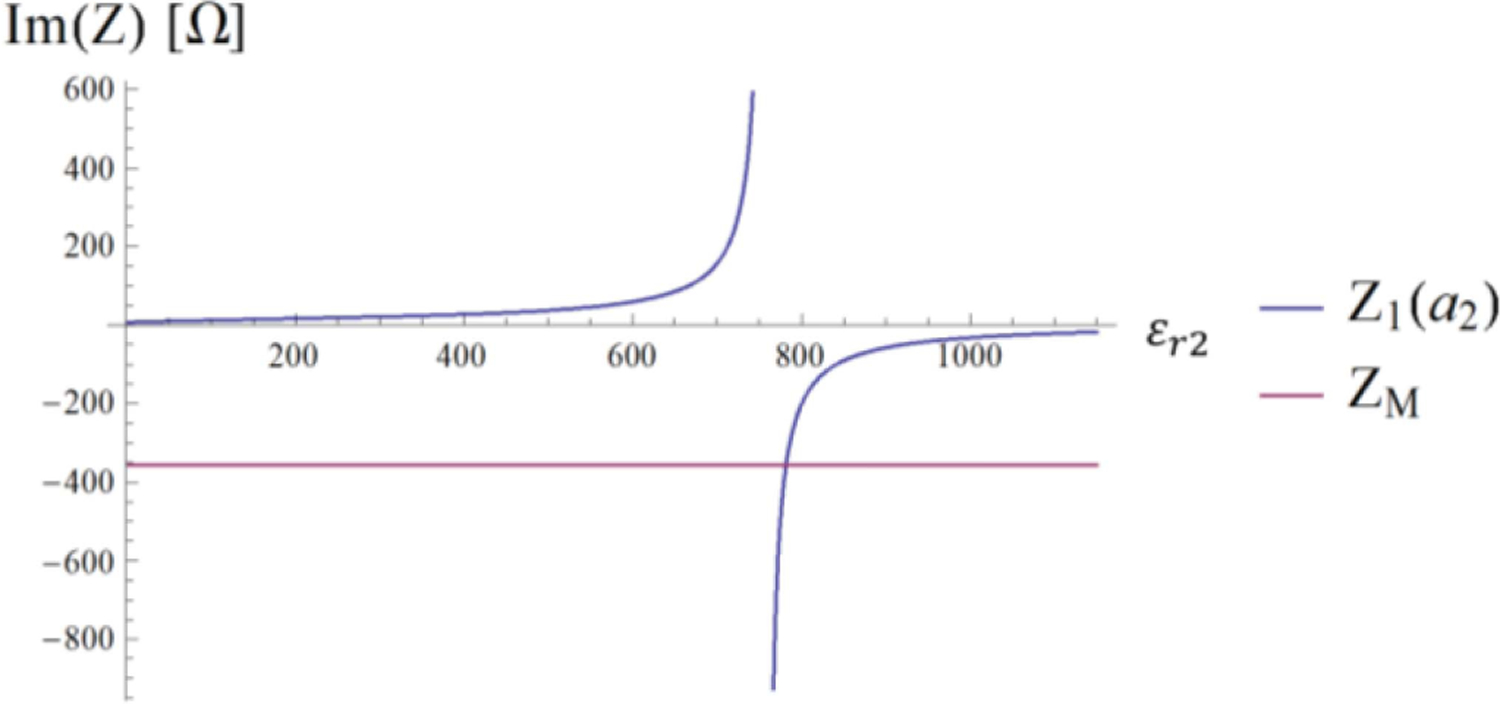
The imaginary part of the impedance *Z*_1_(*a*_2_) seen at the sphere interface is represented in blue. The matching value defined by the second member of equation (16) is represented in magenta.

**Fig. 5.**
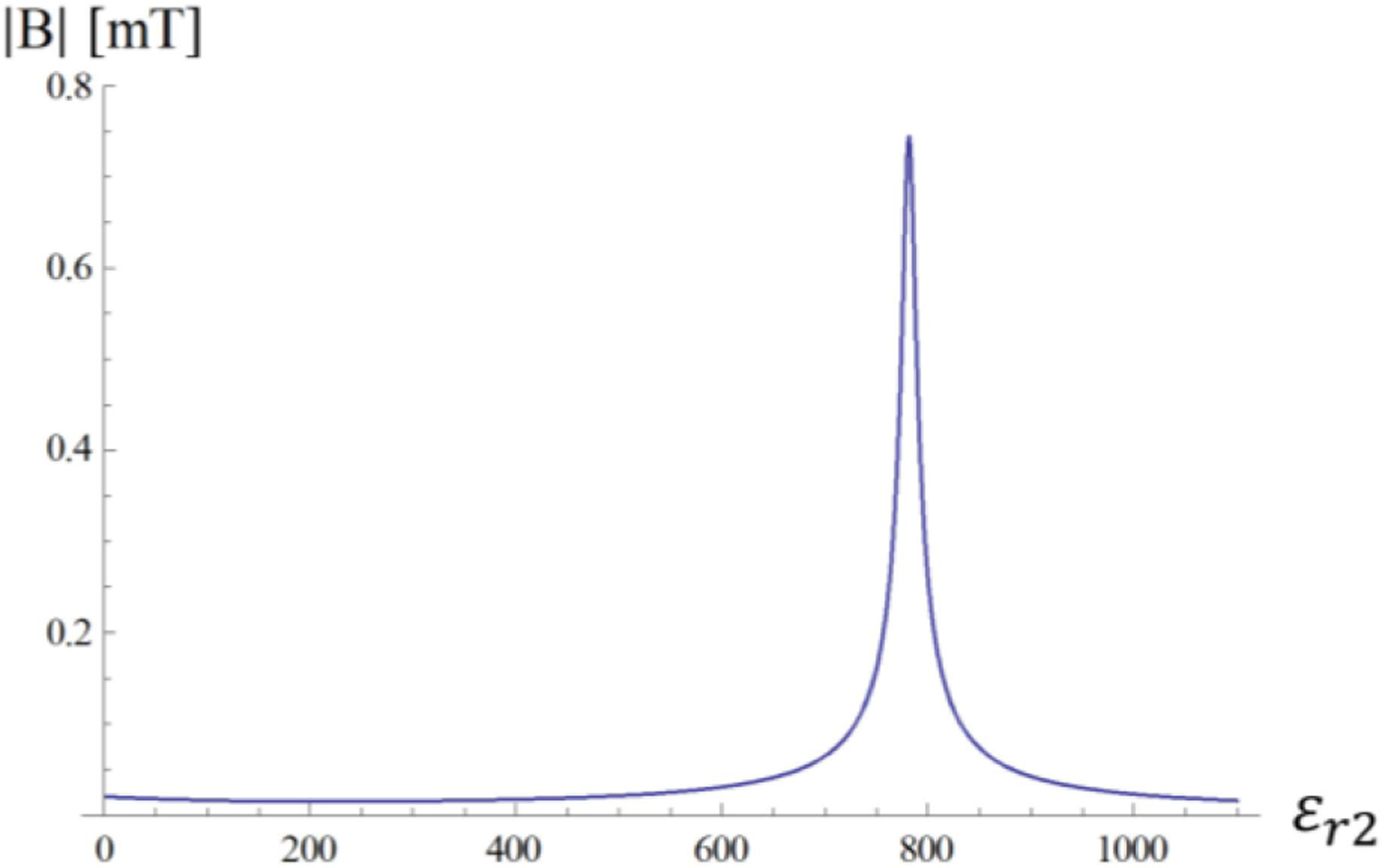
Fundamental mode (n = 1) Transversal Magnetic field at the origin as a function of HPM permittivity. The maximum value is reached at *ε*_*r*2_ = 781.5.

Equation (19) is graphically represented in Fig. 4, where the two curves intersect at *ε*_*r*2_ = 781.5, which corresponds to the peak of the *τ*_12_ coefficient in Fig. 3. Fig. 5 shows the transverse magnetic field at the origin of the sphere as a function of the dielectric constant of the HPM layer.

**Fig. 6.**
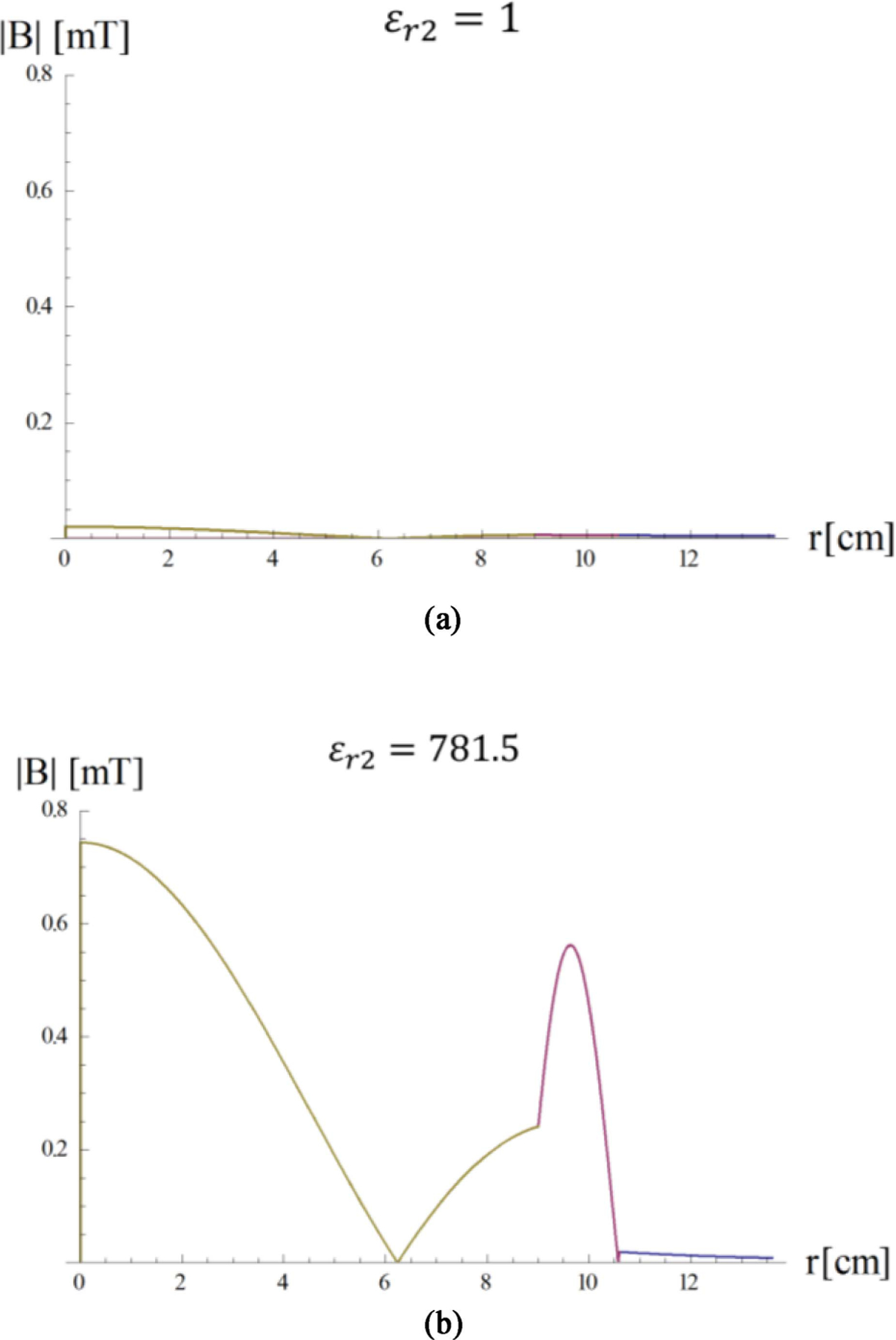
Transversal magnetic field of the fundamental mode (*n* = 1) as a function of the radial distance from the sphere center a) without (a) and with (b) a surrounding layer of lossless HPM with dielectric constant that satisfies the resonance condition. Note that the different colors correspond to the layers, as defined in Fig. 1.

As predicted by the impedance matching equation, the field peaks when the reflection coefficient Γ_*n*_(*k*_1_*a*_2_) is real and positive, which corresponds to *ε*_*r*2_ = 781.5. The effect of the resonance can be appreciated by comparing the magnetic field distribution of the fundamental mode in absence and presence of an HPM layer with the optimal permittivity (Fig. 6).

The previous paragraphs described a simple approach, based on an engineering interpretation that resembles impedance matching, to design the HPM layer that maximizes the electric and, as a consequence, the magnetic field of the fundamental mode inside the sample. This method can be easily extended to different modes and a larger number of layers, providing a tool to interpret and optimize the design of HPM.

### B. Second Mode (n = 2) in the Lossless Sphere

Let us now focus on the second mode (*n* = 2). In order to find *ε*_*r*2_ that maximizes the field coefficient, we can impose the scalar condition on the impedances defined in equation (19).

**Fig. 7.**
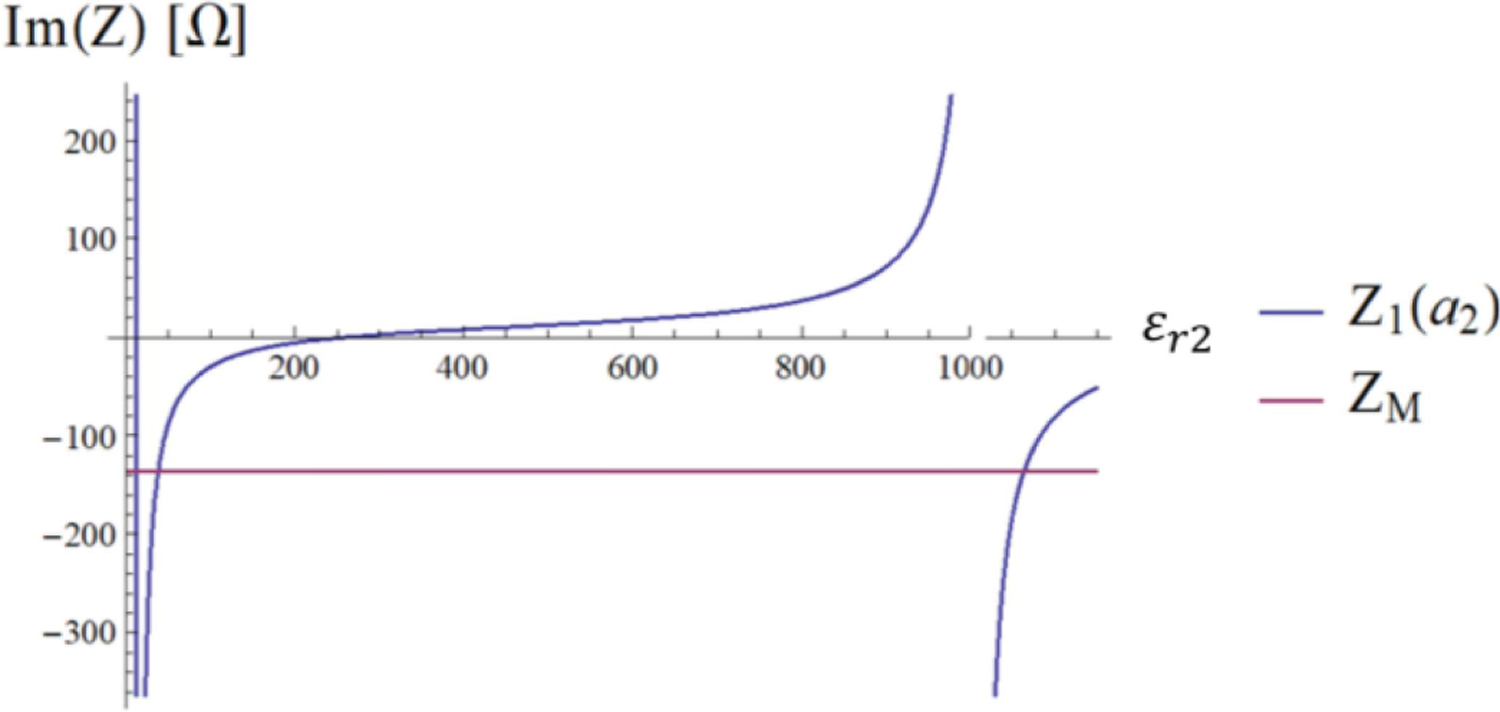
The imaginary part of the impedance *Z*_2_(*a*_2_) seen at the sphere interface is represented in blue. The matching value defined by the second member of equation (19) is represented in magenta.

**Fig. 8.**
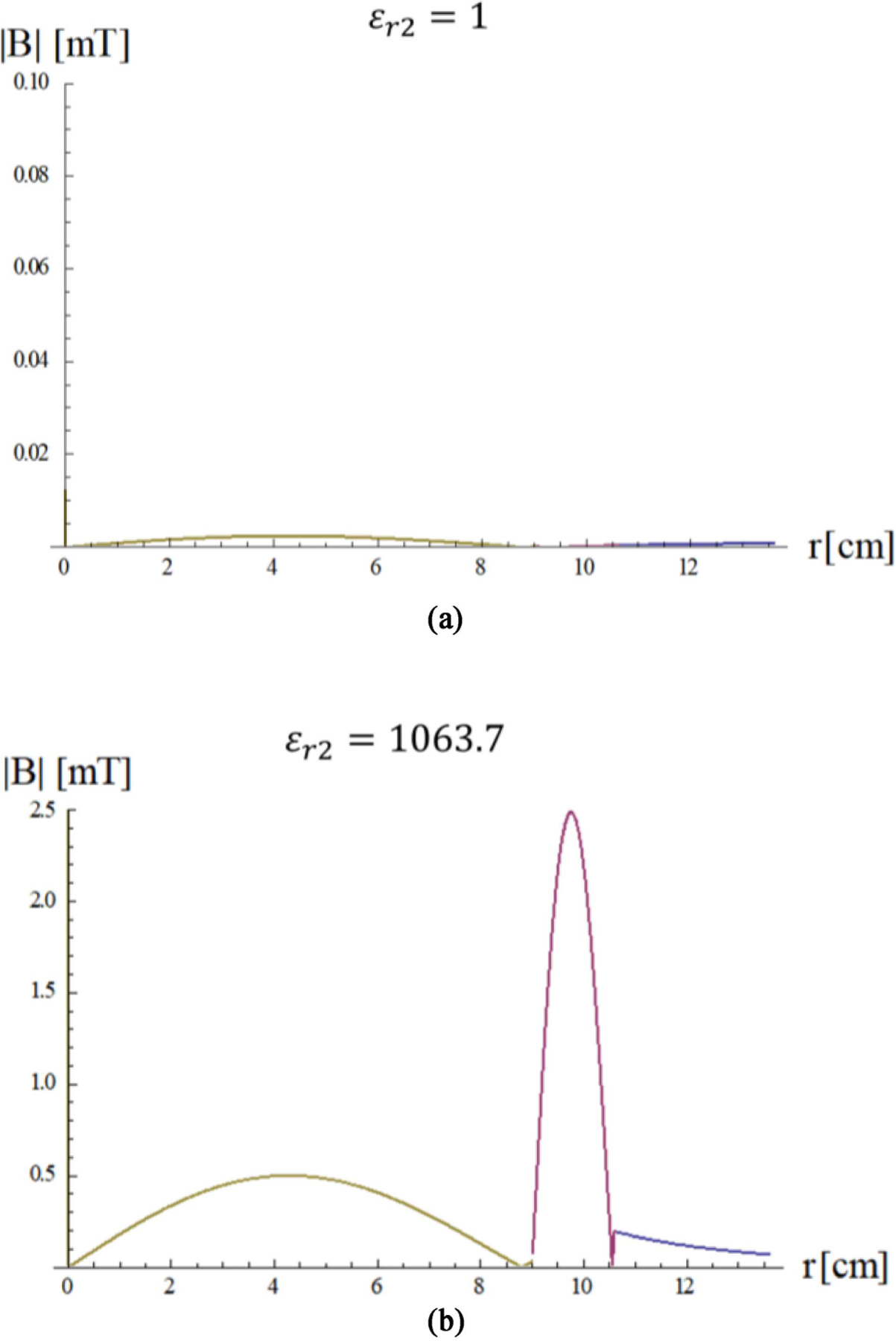
Transversal magnetic field of the second mode (*n* = 2) as a function of the radial distance from the sphere center without (a) and with (b) a surrounding layer of lossless HPM with *ε*_*r*2_= 1063.7.

The graphical representation in Fig. 7 shows that the matching condition is satisfied for *ε*_*r*2_= 37.5 and *ε*_*r*2_= 1063.7. Fig. 8 shows the transverse magnetic field profile associated with the resonance condition established by the latter.

Note that this capacity to selectively enhance a single mode by adjusting the dielectric properties of the HPM is particularly interesting, because the magnetic field peaks at different positions for different modes. For example, the fundamental mode peaks at the origin, whereas the second mode at about *r* = 4 cm. This could be exploited in MRI, for example, to tailor the design of the HPM layer to target specific imaging regions in the head.

Since the relation between the impedance and the HPM permittivity is a multivalued function, it is possible to find a set of *ε*_*r*2_ values that fulfill the matching condition for any mode. In Table II we report the first two solutions (*ε*_*r*2_1_ and *ε*_*r*2_2_) for the first six modes.

### C. Combination of the First Two Modes in the Lossless Sphere

**Fig. 9.**
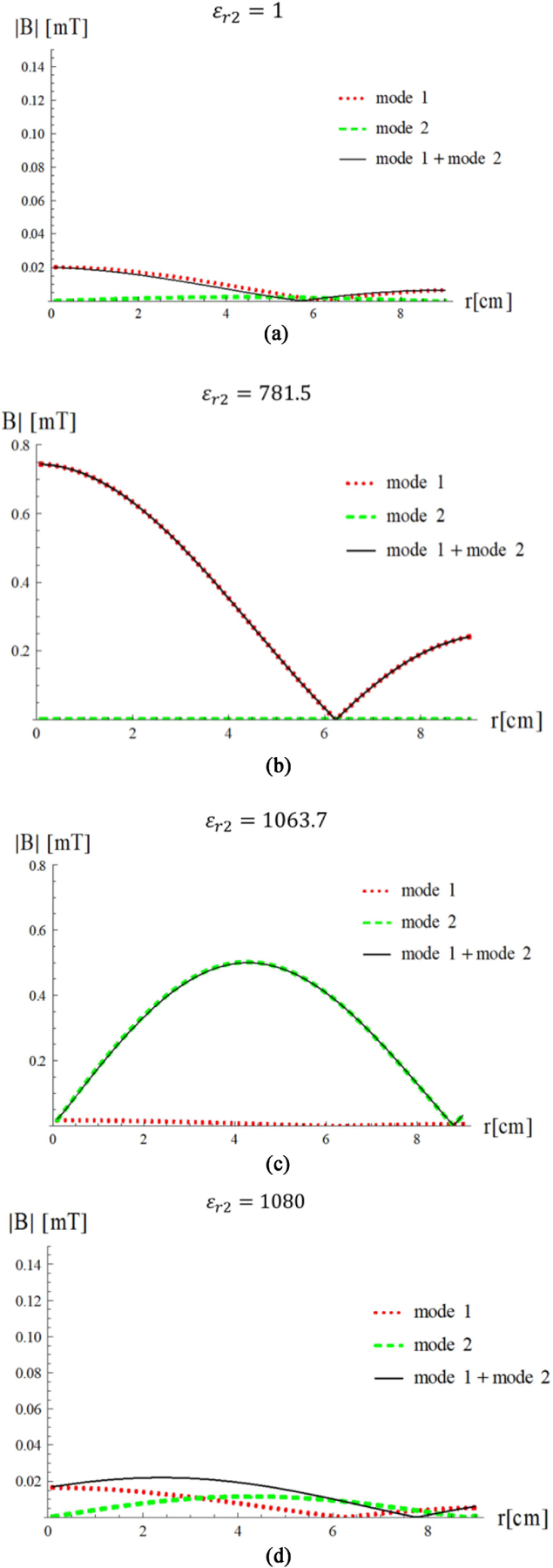
Transversal Magnetic field of the first (red dotted line) and second mode (green dashed line) and their sum (black line) as a function of the distance from the sphere center (a) without HPM and in the presence of a surrounding layer of HPM with (b) *ε*_*r*2_ = 781.5, (c) *ε*_*r*2_ = 1063.7, and (d) *ε*_*r*2_ = 1080.

Let us now assume that the first two modes both propagate inside the uniform sphere. In this case, the choice of *ε*_*r*2_ will change the ratio between the amplitudes of the two modes and, therefore, the overall field distribution. Fig. 9 shows the magnetic field profile for each mode and their sum for different values of the relative permittivity of the HPM layer. In the absence of HPM ( *ε*_*r*2_= 1), the maximum field level is lower than 0.05 mT and the fundamental mode dominates (Fig. 9(a)). Since the magnetic field of the second mode is zero at the origin, if we are interested in imaging near the center of the head, we would try to design an HPM layer that enhance the first mode over the second mode. In fact, the latter would not be sensitive to the MR signal at the position of interest, but would still be sensitive to noise, which is given by the integral of the electric field over the entire volume of the sample [28], therefore affecting the overall SNR. Fig. 9(b) shows that we can obtain this by surrounding the sphere with an HPM layer with *ε*_*r*2_= 781.5, which creates resonance for the fundamental mode, yielding an amplification of more than 30 fold. As a result, almost all the energy is in the center of the sample and the contribution of the second mode to the total field is negligible. If we are interested in imaging a region between the center and the surface of the sample, we could instead use *ε*_*r*2_= 1064, for which the second mode resonates and maximizes the total field at and intermediate position (Fig. 9(c)). If we are instead interested in maximizing field homogeneity over the field of view, we could choose a value of *ε*_*r*2_ that balances the contributions of the two modes (Fig. 9(d)), although with a considerably lower MR signal sensitivity than for the other cases.

### D. Lossy Sphere

**Fig. 10.**
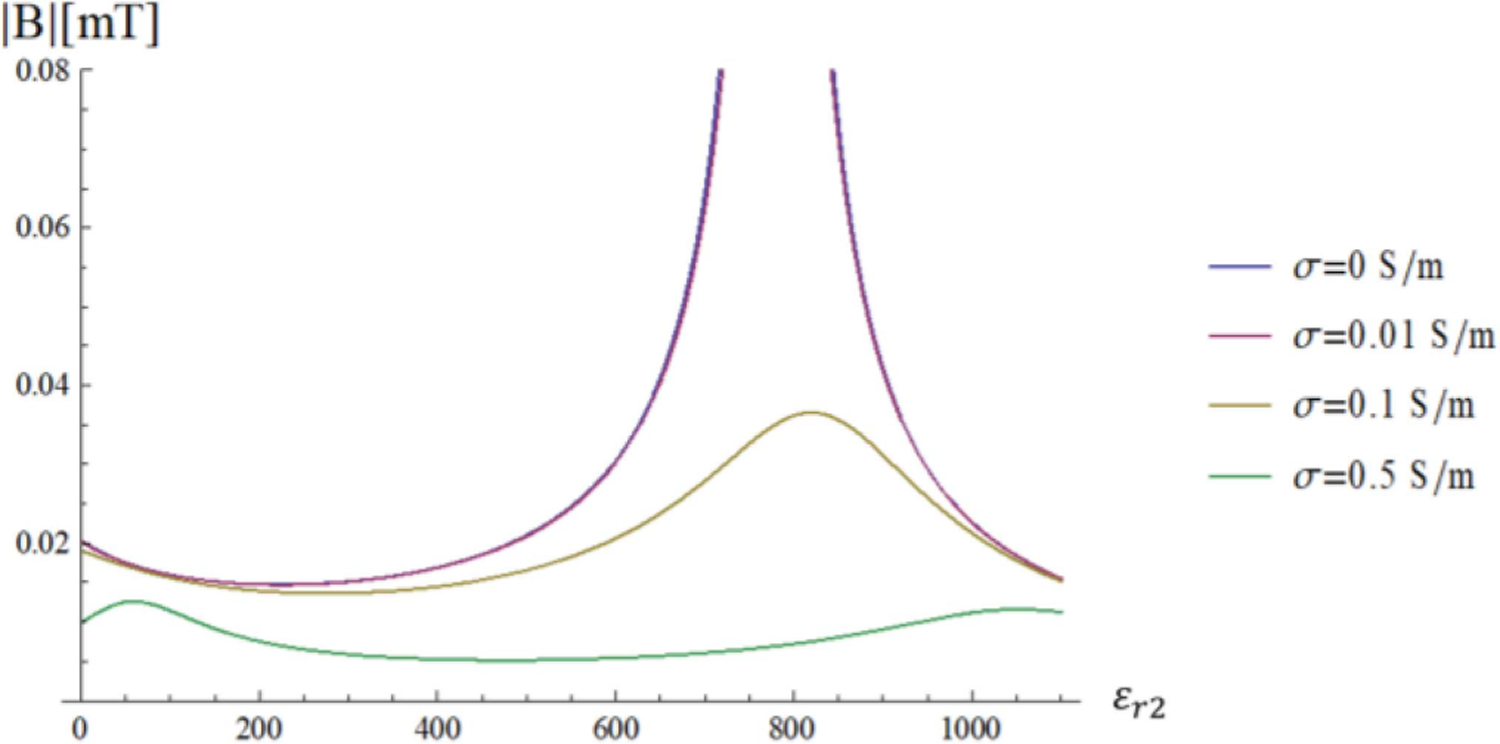
Transversal Magnetic field of the fundamental mode (*n* = 1) at the origin, where it peaks, as a function of the HPM permittivity for different values of the sphere conductivity.

**Fig. 11.**
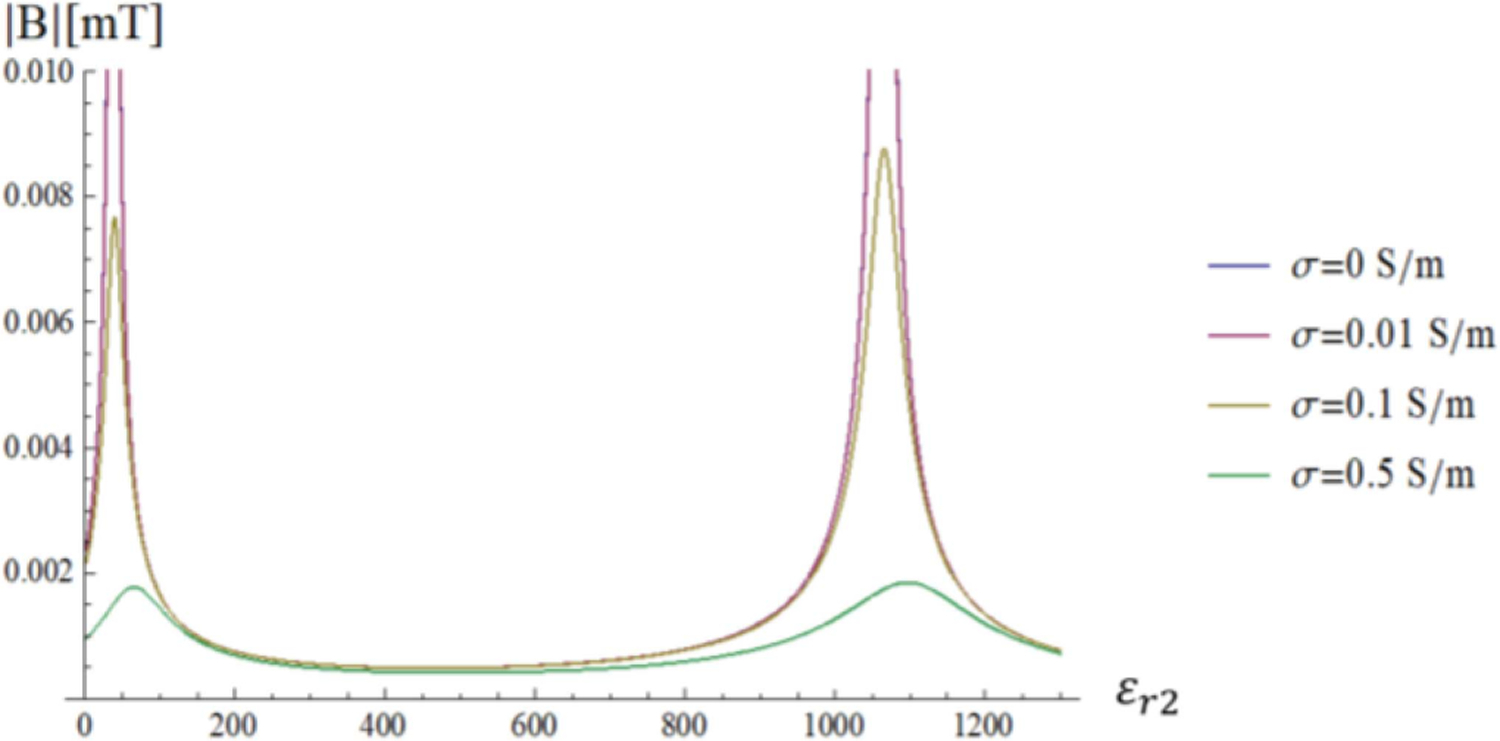
Transversal Magnetic field of the second mode (*n* = 2) at the position where it peaks (4 cm from the origin), as a function of the HPM permittivity for different values of the sphere conductivity.

In practical cases, the sample conductivity *σ*_3_ of the innermost medium is not null and the matching condition in equation (19) cannot be fulfilled for any real value of *ε*_*r*2_, because the sphere impedance is not purely imaginary anymore. However, a condition for which particular modes are amplified can still be found by maximizing the modulus of *τ*_12_ = 1 + Γ_*n*_(*k*_1_*a*_2_), which occurs when the reflection coefficient Γ_*n*_(*k*_1_*a*_2_) is real and positive. Fig. 10 shows the transverse magnetic field of the fundamental mode at the origin of the sphere as a function of the dielectric constant of the HPM layer for different values of the sphere conductivity (*σ*_3_ = 0, 0.01, 0.1 and 0.5 S/m). Fig. 11 shows the transverse magnetic field of the second mode for the same cases, but at a distance of 4 cm from the origin of the sphere. In both instances, when the sample conductivity is not negligible, as in most biological tissues, the value of the optimal *ε*_*r*2_ for the HPM is shifted compared to the lossless case, because a different phase matching between inward and outward waves is required.

**Fig. 12.**
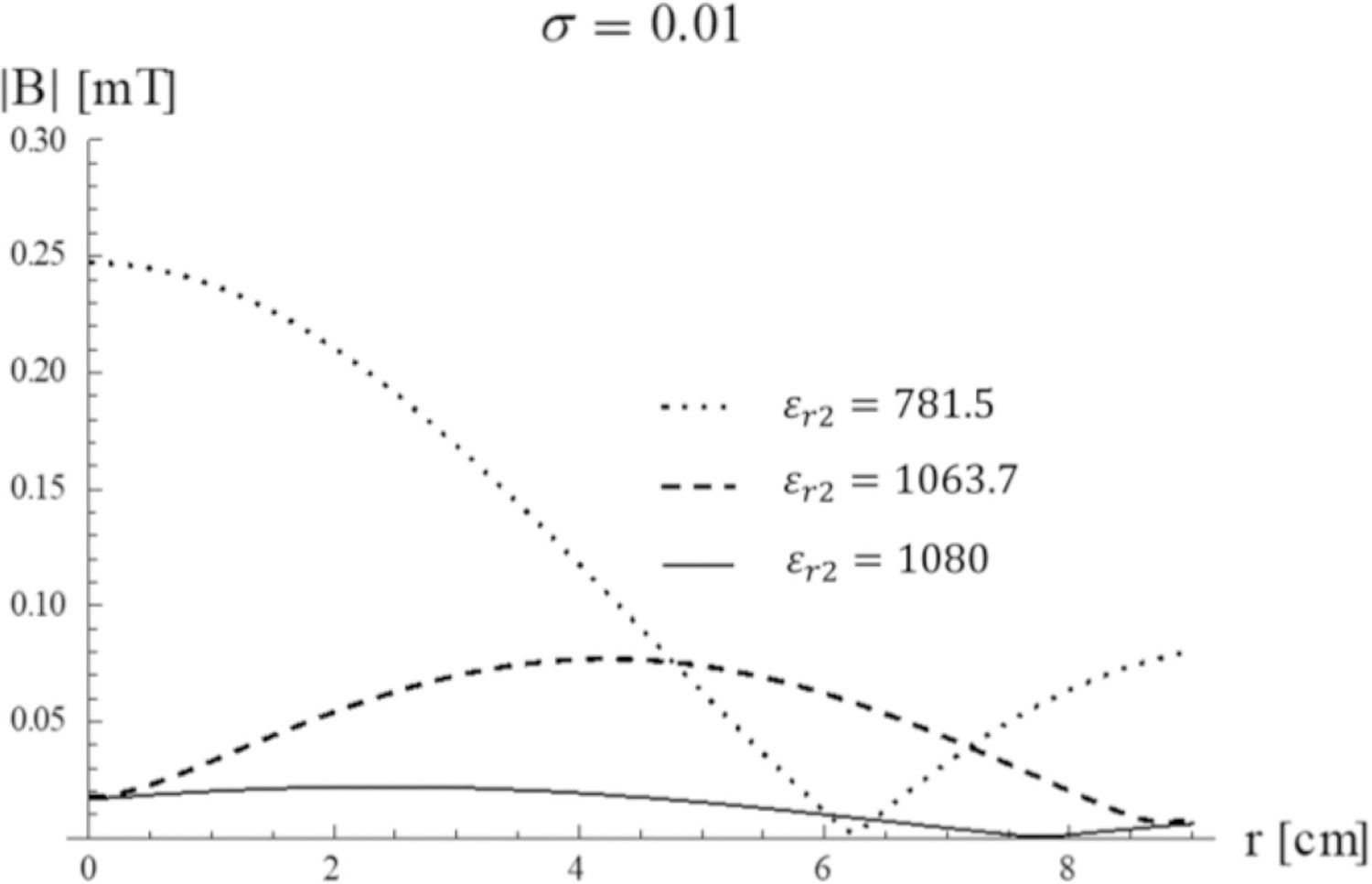
Transversal Magnetic field of the first two modes as a function of the distance from the sphere center for different *ε*_*r*2_ values. The inner sphere conductivity is *σ*_3_= 0.01 S/m.

**Fig. 13.**
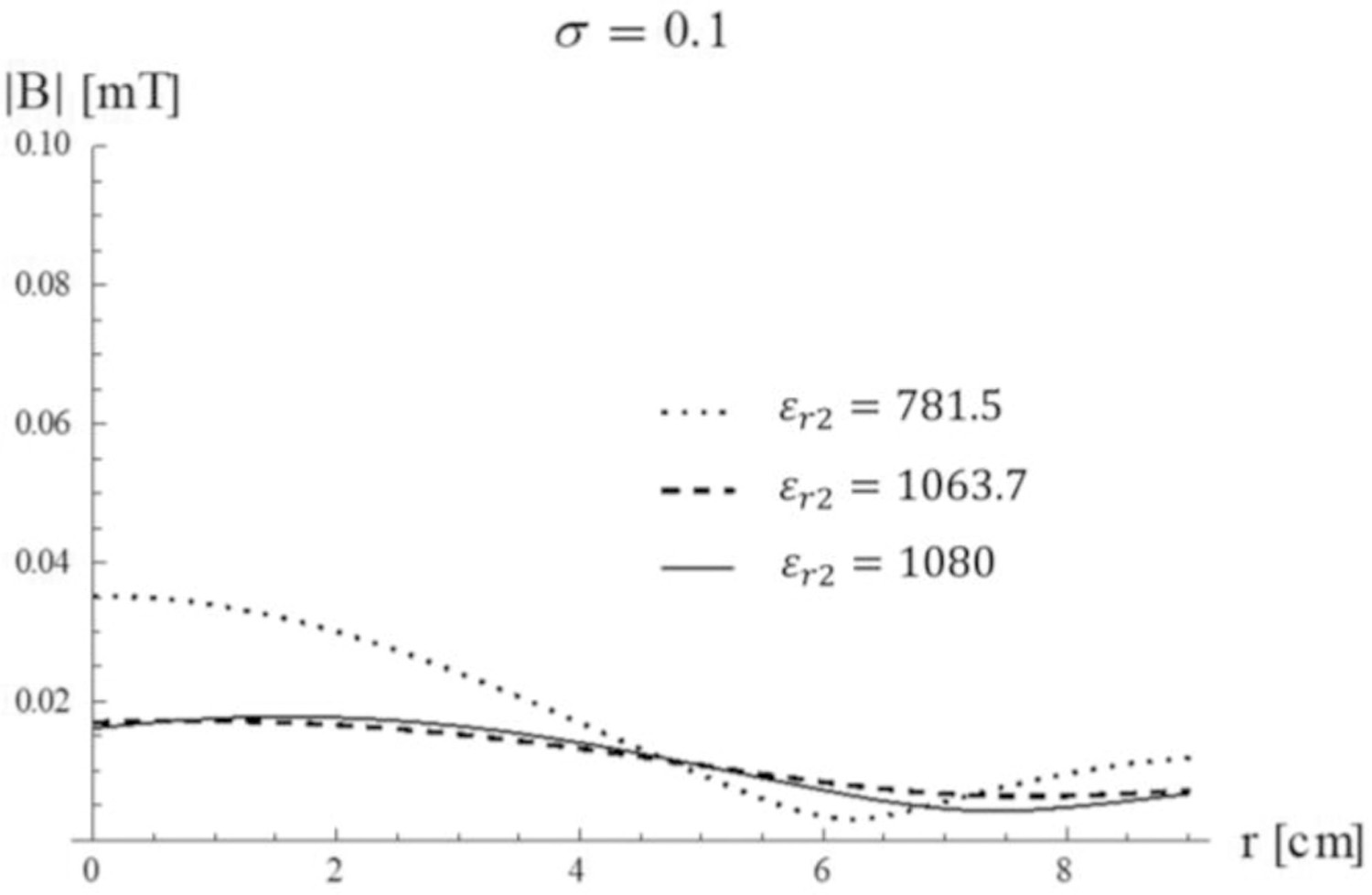
Transversal Magnetic field of the first two modes as a function of the distance from the sphere center for different *ε*_*r*2_values. The inner sphere conductivity is *σ*_3_= 0.1 S/m.

When the conductivity of the sample is small (for example, for *σ*_3_ = 0.01 S/m), the *ε*_*r*2_ values that selectively amplify the first two modes are the same of the lossless case. Therefore, the field distribution is similar, although the maximum amplitude of the field is considerably lower (Fig. 12 *vs.* Fig. 9). When the conductivity of the sample is larger (for example, for *σ*_3_ = 0.1 S/m), the second mode does not dominate the overall behavior anymore for *ε*_*r*2_ = 1063.4, but it is attenuated and yields approximately the same homogeneous field distribution as for *ε*_*r*2_ = 1080 (Fig. 13).

**Fig. 14.**
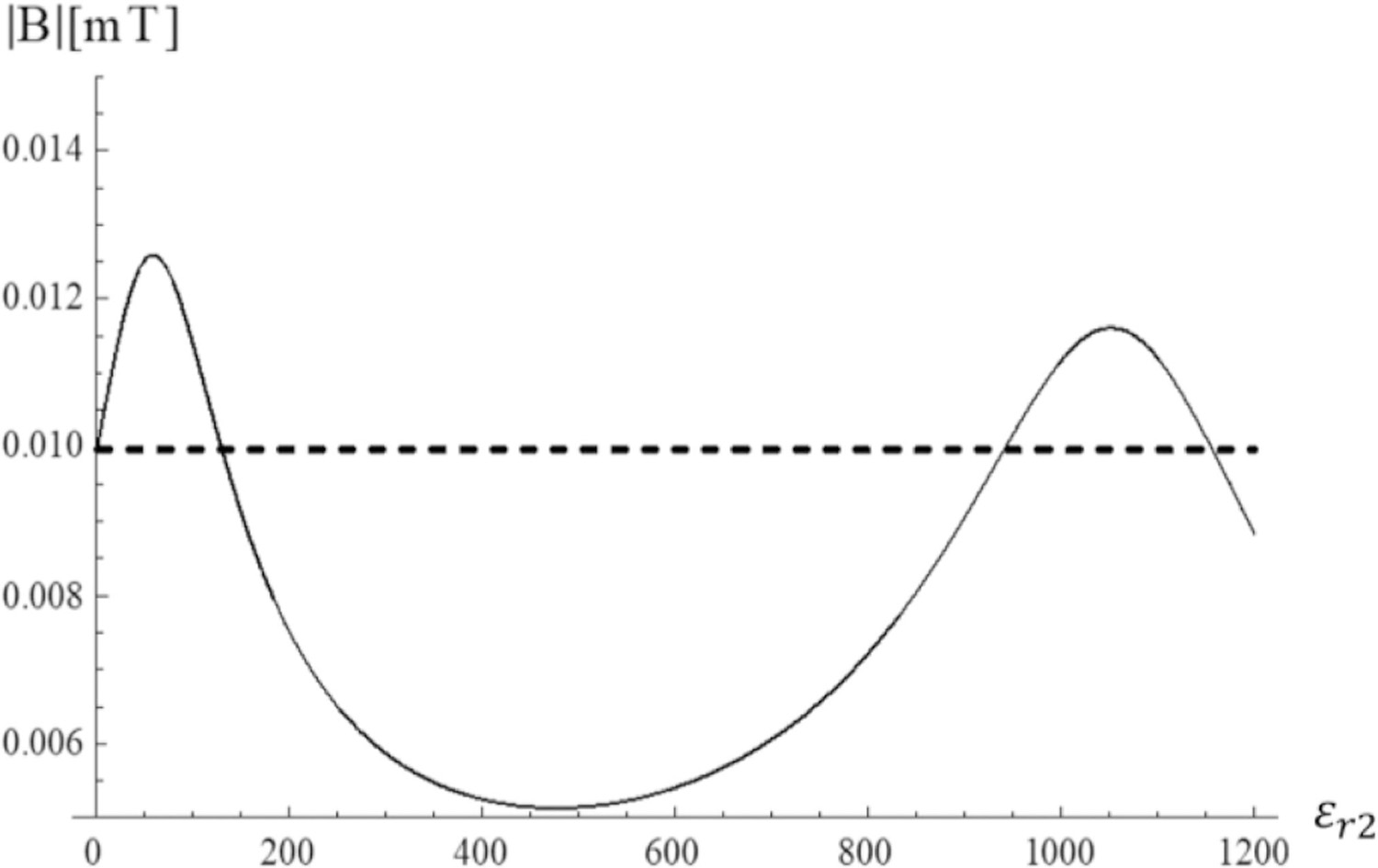
Fundamental mode (n = 1) Transversal Magnetic field at the origin as a function of HPM permittivity for *σ*_3_ = 0. 5 S/m. The dashed line represents the value of the field taken in the absence of the HPM.

The case of *σ*_3_ = 0.5 S/m is of particular interest for coil design, because, combined with the *ε*_*r*3_ = 50 used throughout this work, it mimics the average electrical properties of the human brain at 7 T [28] Fig. 14 shows the transverse magnetic field for the fundamental mode at the origin of the sphere as a function of the dielectric constant of the HPM layer when *σ*_3_ = 0.5 S/m (rescaled version of one of the curves in Fig. 10). In the absence of HPM ( *ε*_*r*2_ = 1), the central magnetic field is 0.01 mT (dashed line). The field peaks at 0.126 mT for *ε*_*r*2_ = 60 and its value is at least 20% larger than in the case without HPM for *ε*_*r*2_ between 20 and 100.

## IV. Discussion

We introduced a framework to analyze the radial dependence of the transverse field inside a uniform sphere on the dielectric constant of a surrounding HPM layer. We used simple canonical cases to show that the field inside the sample can be manipulated via HPM design. In the MRI literature, the effect of HPM on the RF field distribution has been explained with different arguments. The most adopted one is based on the Maxwell-Ampere law, for which HPM generate high displacement currents, inducing secondary fields that increase SNR [16], [17], [33], [34]. While secondary fields are certainly contributing, our results show that the role of HPM is more complex. Figs 2 to 4 show that properly designing the HPM can make a single mode resonate by maximizing the transmission coefficient *τ*_12_. In other words, the HPM not only introduces secondary fields, but reshapes the fields of each mode, acting on the combination of amplitudes and phases of ingoing and outgoing waves.

Previous work suggested that the HPM permittivity that maximizes transmit efficiency is the one that optimizes near-field wave impedance matching between the power source and the sample [35].

This work also shows that with a proper combination of HPM thickness and relative permittivity, the phase of ingoing and outgoing fields can be matched at the HPM-air interface, providing a stationary resonant wave in the sample that amplifies performance. All the other modes that are not in resonance yield much lower field amplitudes.

This interpretation based on the resonance condition explains why, neither in electromagnetic simulations nor in experiments, a monotonic increase of the HPM relative permittivity corresponds to a linear increase of the SNR, as it would be expected if only secondary fields were responsible for the enhanced coil performance.

The secondary field argument does not explain either why HPM can increase SNR or decrease SAR, which would mean that somehow the secondary magnetic field dominates over the secondary electric field. On the other hand, our framework shows that HPM could be designed to enhance only the modes that contribute to the magnetic field at a target location, cutting off the remaining modes that would only contribute to the overall image noise or RF energy deposition, in the receive or transmit case, respectively.

One practical difficulty in optimizing HPM for MRI is that the optimal permittivity depends on the problem geometry. For example, in the case of the spherical geometry adopted in this work, the fields depend on the product between the medium wavenumber *k* and the radial coordinate *r.* Therefore, the overall effect of the HPM is determined by the combination of the geometry and the dielectric constant of the HPM layer, which influences the wavenumber value. This suggests that for different sample geometry and properties, the thickness and the relative permittivity of the HPM substrate should be jointly optimized.

Our analytical framework could be extended to a body-mimicking geometry, such as the cylinder. A uniform cylinder with average body electrical properties was successfully employed to approximate the human body in MRI simulations [13], [36], providing useful physical insight to optimize coil design [37], [38]. To replicate this work for applications in body imaging, one would need to implement a multi-layered cylindrical model, for example following the theory in [39]. While our formalism would be readily applicable, we expect a different behavior than for the sphere case, due to differences in symmetry of the geometry and electrical properties of the tissue-mimicking layers. For example, a thick layer of fat near the HPM layer could considerably affect the propagation of the EM field.

Our formalism enables one to investigate the effect of HPM for different radii and relative permittivity values of the spherical sample, and the obtained results demonstrate that the proposed framework is a useful tool to physically interpret the effect of HPM on coil performance in MRI. Note that the use of a complete basis [40] instead of actual coils is not limiting the validity of our observations, since finite coils can be approximated by linear combinations of the basis functions used in this work [37], [41]. For example, the first mode (*n* = 1) can closely approximate the behavior of MRI coils, such as the birdcage coil [42], that are designed to achieve quadrature volume excitation to maximize performance at the center of the head [28], [37], [42].

Our results for the lossless sphere show that the resonance condition occurs over a narrow interval of *ε*_*r*2_ values, which could make practical applications challenging. However, Fig. 10 suggests that, in the case of a lossy sphere, although the selective mode amplification is reduced due to the damping, a wider range of *ε*_*r*2_ could yield a homogeneous field distribution, which is also desirable at UHF MRI.

For *ε*_*r*2_ ranging between 20 and 100, the field inside the lossy sphere was larger by 20% or more than in the case without HPM (Fig. 14). This confirms that even in the case of a lossy sample, a substantial performance gain can be achieved in deep regions, in addition to the superficial gain provided by the secondary fields generated by the displacement currents. In fact, relative permittivity of 100 for the HPM yielded improved central and peripheral SNR for brain imaging at 7 T in a recent experimental work [17]. The same value was also found to be nearly optimal at 7 T by previous simulation studies using either a similar spherical geometry [16], [43], or realistic head models [44]. Fig. 14 also shows that choosing a suboptimal permittivity for the HPM could hurt efficiency, which was also suggested by previous work [43].

Finally, note that in this work we kept a fixed thickness for HPM layer and the optimal value of *ε*_*r*2_ is expected to increase or decrease for thinner or thicker HPM, respectively [16].

## V. Conclusion

In this paper, we presented a framework for interpreting the effect of HPM on coil performance in MRI. We employed a multi-layered spherical model and focused on the radial dependence of the field, which is the only one affected by the dielectric constant of the HPM. We showed that a HPM layer surrounding a sample can act as a filter to selectively enhance individual modes in order to shape the field distribution and increase SNR at specific locations. Our formulation provides an explanation of the mode amplification in terms of matching, enabling the estimation of suitable dielectric constants for the HPM with a simple mode-matching formula.

## Figures and Tables

**TABLE I:** Definitions of the Different Wave Impedances (from [18])

Spherical Bessel Function	Impedance Expression	Impedance Symbol	Compact Expression
$h_{n}^{\left(1\right)}\left(k_{l}r\right)$	$\frac{i\omega \mu }{k_{l}}\frac{h_{n}^{\left(1\right)}\left(k_{l}r\right)}{h_{n}^{{\left(1\right)}^{\prime }}\left(k_{l}r\right)}$	$Z_{n}^{\left(1\right)}\left(k_{l}r\right)$	$Z_{nl}$
$h_{n}^{\left(2\right)}\left(k_{l}r\right)$	$\frac{i\omega \mu }{k_{l}}\frac{h_{n}^{\left(2\right)}\left(k_{l}r\right)}{h_{n}^{(2{)}^{\prime }}\left(k_{l}r\right)}$	$Z_{n}^{\left(2\right)}\left(k_{l}r\right)$	$\bar{Z_{nl}}$
$j_{n}\left(k_{l}r\right)$	$\frac{i\omega \mu }{k_{l}}\frac{j_{n}\left(k_{l}r\right)}{j_{n}^{\prime }\left(k_{l}r\right)}$	$Z_{n}^{\left(J\right)}\left(k_{l}r\right)$	$Z_{Jnl}$

**TABLE II:** First Two Values of the Relative Dielectric Constants That Maximize the Field Associated with Modes 1 to 6

Mode n.	*ε*_*r*2_1_	*ε*_*r*2_2_
1	781.5	3781
2	37.5	1063.7
3	106	1183
4	164	1282
5	222	1377
6	281	1471
